# Draft genome sequences and antimicrobial susceptibility profiles of one K1 and five K2 clinical hypervirulent *Klebsiella pneumoniae* isolates from Ghana

**DOI:** 10.1128/mra.00887-26

**Published:** 2026-10-22

**Authors:** William Boateng, Christian Owusu-Nyantakyi, Grebstad Rabbi Amuasi, Blessing Kofi Adu Tabi, Emmanuel Ohene Kyei McKeown, Agnes Oclu, Justice Danso, Quaneeta Mohktar, Alfred Bortey, Felicia Owusu, Daniel Baka, Gabriella Acquah, Maa Lankai Quaye, Angella Ama Yayra Kampo, Solomon Asante-Sefa, Ebenezer Kofi Mensah, Francis Kwame Morgan Tetteh, Rhodalyn Tagoe, Bright Adu, Beverly Egyir

**Affiliations:** 1 Department of Bacteriology, Noguchi Memorial Institute for Medical Research, University of Ghana 58835, Accra, Ghana; 2 Sekondi Zonal Public Health Laboratory, Regional Health Directorate, Ghana Health Service, Sekondi-Takoradi, Ghana; 3 Pathology Division, 37 Military Hospital, Accra, Ghana; 4 Department of Immunology, Noguchi Memorial Institute for Medical Research, University of Ghana 58835, Accra, Ghana; Nanchang University, Nanchang, Jiangxi, China

**Keywords:** Hypervirulence, *Klebsiella pneumoniae*, K1, K2, hvKP, Ghana, LMIC

## Abstract

We report the draft genome sequences of six clinical hypervirulent *Klebsiella pneumoniae* isolates from Ghana. These isolates carry critical hypervirulence determinants, including siderophores and hypermucoidy regulators. These genomic sequences significantly enrich the West African data set on hypervirulent *K. pneumoniae*, providing vital baselines to support the surveillance of emerging high-risk lineages.

## ANNOUNCEMENT

Hypervirulent *Klebsiella pneumoniae* (hvKP) causes severe community- and hospital-acquired infections worldwide, (1) but genomic data from Africa remain limited, constraining understanding of its molecular epidemiology and dissemination (2). Here, we report the draft genome sequences of six clinical hvKP isolates recovered in Ghana to support regional genomic surveillance. *K. pneumoniae* isolates (*n* = 201), recovered by routine culture, Gram staining, and biochemical identification of clinical specimens collected at ten hospitals across Ghana between 2020 and 2022, were transported to Noguchi Memorial Institute for Medical Research for further analysis. The six isolates reported here were selected following genomic screening because they carried hypervirulence-associated determinants. The stored isolates were cultured on MacConkey agar (Oxoid, UK) plates and incubated at 37°C for 18–24 h. Resulting colonies were sub-cultured on Nutrient agar (Oxoid, UK) plates and incubated at 37°C for 18–24 h. Species-level identification was subsequently confirmed via matrix-assisted laser desorption ionization-time of flight mass spectrometry (MALDI-TOF MS) (Bruker Daltonics, Germany). Antimicrobial susceptibility testing was performed by the Kirby-Bauer disk diffusion method using 13 antimicrobial agents (Oxoid, UK) on Mueller-Hinton agar (Oxoid, UK) plates with a 0.5 McFarland inoculum and incubation at 37°C for 18–24 h. *Escherichia coli* ATCC 25922 was used for quality control, and measured zone sizes were interpreted using CLSI guidelines (2025) (3). The isolates were cultured on Nutrient agar (Oxoid, UK) plates and incubated for 18–24 h at 37°C. Subsequently, DNA extraction and purification were performed using the QIAamp DNA Minikit (Qiagen, Germany). Quantification of DNA was done using a Qubit 4.0 fluorometer and the High Sensitivity 1X dsDNA Assay Kit (Thermo Fisher Scientific, USA). Libraries were prepared using the Illumina DNA Preparation (M) Tagmentation Kit (Illumina Inc., USA) according to the manufacturer’s instructions. The process involved simultaneous DNA fragmentation and adapter tagging via bead-linked transposons, followed by indexing PCR and bead-based size selection. Library fragment sizes and concentrations were assessed using the Agilent 2100 Bioanalyzer (Agilent Technologies, USA) and quantitative PCR (KAPA SYBR Fast qPCR Kit), respectively. Libraries were normalized to 2 nM, pooled, and then loaded on a 2 × 150 bp cycle P3 cartridge for sequencing on the Illumina NextSeq 2000 system (Illumina Inc., USA). Indexes and reads with Phred quality scores <20 were trimmed using Trimmomatic v0.39 (4), quality-checked with FastQC v0.11.9 (5), and assembled using Unicycler v0.5.0 (6) in short-read-only mode. Contigs <200 bp were removed, and assembly statistics were calculated using QUAST v5.2.0 (7) with the 200 bp cutoff. CheckM v1.1.6 (8) was used to evaluate the completeness and contamination of assembled genomes. The assemblies were analyzed using PathogenWatch (accessed 14 July 2026) (9) through its integrated Kleborate workflow to verify the species, determine serotype, multilocus sequence type, hypermucoidy-associated genes, resistance, and virulence factors. ResFinder v4.7.2 (https://genepi.food.dtu.dk/resfinder) (10) was used to screen the assemblies further for resistance determinants. Genome annotation was performed using NCBI PGAP v6.3 (11). Default settings were used except where mentioned. Table 1 presents a summary of the results.

**TABLE 1:** Antimicrobial resistance profiles and genomic characteristics of isolates^*a*^^,*b*^

Characteristics	Isolate information					
Sample ID	SPHL_029_S215	SPHL_042_S229	SPHL_033_S213	SPHL_040_S231	SPHL_039_S221	KP25_S12
Strain ID	KP0192	KP0200	KP0196	KP0198	KP0197	KP0167
Specimen	Sputum	Sputum	Sputum	Sputum	Sputum	Wound
Sentinel site	Effia Nkwanta Hospital	Effia Nkwanta Hospital	Effia Nkwanta Hospital	Effia Nkwanta Hospital	Effia Nkwanta Hospital	37 Military Hospital
City	Sekondi	Sekondi	Sekondi	Sekondi	Sekondi	Accra
Geographical coordinates	4.92438° N, 1.74256° W	4.92438° N, 1.74256° W	4.92438° N, 1.74256° W	4.92438° N, 1.74256° W	4.92438° N, 1.74256° W	5.58691° N, 0.18471° W
Antimicrobial agents (Zone diameters/mm)						
Amikacin (30 µg)	S (22)	S (23)	S (24)	S (21)	S (22)	S (22)
Gentamicin (10 µg)	S (21)	S (20)	S (22)	S (20)	S (19)	S (21)
Ampicillin (10 µg)	R (6)	R (6)	R (10)	R (6)	R (6)	R (6)
Piperacillin-Tazobactam (100/10 µg)	S (26)	S (26)	S (28)	S (26)	S (25)	S (25)
Cefotaxime (30 µg)	S (34)	S (33)	S (35)	S (31)	S (34)	S (34)
Cefoxitin (30 µg)	S (21)	S (22)	S (21)	S (21)	S (21)	S (21)
Cefepime (30 µg)	S (35)	S (34)	S (35)	S (32)	S (33)	S (34)
Cefuroxime (30 µg)	S (24)	S (23)	S (26)	S (22)	S (24)	S (23)
Chloramphenicol (30 µg)	S (25)	S (25)	S (28)	S (26)	R (6)	S (27)
Ciprofloxacin (5 µg)	S (28)	S (27)	S (30)	S (30)	S (29)	S (29)
Meropenem (10 µg)	S (32)	S (32)	S (32)	S (32)	S (32)	S (33)
Tetracycline (30 µg)	S (20)	S (21)	S (21)	S (21)	S (22)	S (23)
Trimethoprim-Sulfamethoxazole (25 µg)	S (24)	R (6)	S (24)	S (26)	R (6)	S (26)
Resistance genes						
Aminoglycosides	ND	ND	*strB*, *strA, aph (6)-Id, aph(3'')-Ib*	ND	ND	ND
Fluoroquinolones	*OqxA, OqxB*	*OqxA, OqxB*	*OqxA, OqxB*	*OqxA, OqxB*	*OqxA, OqxB*	*OqxA, OqxB*
Fosfomycin	*fosA6*	*fosA6*	*fosA6*	*fosA*	*fosA*	*ND*
Penicillins	*bla*_SHV-11_	*bla*_SHV-28_	*bla*_SHV-11_	*bla*_SHV-1_	*bla*_SHV-1_	*bla*_SHV-1_
Phenicols	*catA1, OqxA, OqxB*	*OqxA, OqxB*	*OqxA, OqxB*	*OqxA, OqxB*	*OqxA, OqxB*	*OqxA, OqxB*
Sulfonamides	*sul1*	ND	*sul2*	ND	ND	ND
Trimethoprim	*dfrA15, OqxA, OqxB*	*OqxA, OqxB*	*dfrA14, OqxA, OqxB*	*OqxA, OqxB*	*OqxA, OqxB*	*OqxA, OqxB*
Virulence genes	*iuc1*, *iro1*	*iuc1, iro1*	*iuc1*, *iro1*	*ybt1, iuc1, iro1*	*ybt9, iuc1, iro1*	*iuc1, iro1*
Hypermucoidy regulating genes	*rmp1*, *rmpA2_5*	*rmp1*, *rmpA2_6*	*rmp1*, *rmpA2_5*	*rmp1*	*rmp1, rmpA2_9*	*rmp1, rmpA2_9*
Virulence score	3	3	3	4	4	3
Plasmids	IncFIA(pBK30683)	repB	IncHI1B(pNDM-MAR), repB	IncHI1B(pNDM-MAR), repB	IncHI1B(pNDM-MAR), repB, Col440I	IncHI1B(pNDM-MAR), repB
Multilocus sequence type	25	2159	65	86	86	86
K Locus	KL2	KL1	KL2	KL2	KL2	KL2
K Serotype	K2	K1	K2	K2	K2	K2
O Locus	OL2α.2	OL2α.2	OL2α.2	OL2α.1	OL2α.1	OL2α.1
O Serotype	O1αβ,2β	O1αβ,2β	O1αβ,2α	O1αβ,2α	O1αβ,2α	O1αβ,2α
Genome size (bp)	5,593,125	5,354,293	5,272,894	5,420,728	5,411,502	5,470,201
N_50_ (bp)	257,951	333,437	171,803	320,439	362,234	296,728
GC content (%)	57.2	57.4	57.3	57.3	57.3	57.0
No. of reads	3,943,620	6,945,938	5,029,188	3,758,222	5,802,760	3,268,472
No. of contigs	123	51	87	51	43	51
Size of largest contig (bp)	538,194	723,870	597,286	516,714	623,361	501,833
No. of coding sequences	5,528	5,143	5,078	5,190	5,170	5,293
No. of rRNAs	7	4	4	3	3	3
No. of tRNAs	78	76	77	77	78	77
Coverage (×)	96	176	130	94	146	81
Completeness (%)	100	100	100	100	100	100
Contamination (%)	1.2	0.7	1.4	1	0.7	1.1
Genome accession no.	JAPNYM010000000	JAPNYE010000000	JAPNYI010000000	JAPNYG010000000	JAPNYH010000000	JAPNZL010000000
Genbank assembly	GCF_026671405.1	GCF_026671415.1	GCF_026672145.1	GCF_026671555.1	GCF_026671615.1	GCF_026674255.1
BioSample accession no.	SAMN31892426	SAMN31892434	SAMN31892430	SAMN31892432	SAMN31892431	SAMN31892401
SRA accession no.	SRR37646312	SRR37646311	SRR37646310	SRR37646309	SRR37646308	SRR39231029

^*a*^ R, resistant; S, susceptible; ND, not detected.

^*b*^ All isolates were recovered in 2022.

## Data Availability

The assembled data were uploaded to the National Center for Biotechnology Information database and assigned the BioProject accession number PRJNA906096. The whole-genome sequencing project was deposited in DDBJ/ENA/GenBank under the accession numbers as provided in Table 1.
